# Crystal structure of [2,13-bis­(acetamido)-5,16-dimethyl-2,6,13,17-tetra­aza­tri­cyclo­[16.4.0.0^7,12^]docosane-κ^4^ *N*]silver(II) dinitrate from synchrotron X-ray data

**DOI:** 10.1107/S2056989018003560

**Published:** 2018-03-06

**Authors:** Dohyun Moon, Jong-Ha Choi

**Affiliations:** a Pohang Accelerator Laboratory, POSTECH, Pohang 37673, Republic of Korea; b Department of Chemistry, Andong National University, Andong 36729, Republic of Korea

**Keywords:** crystal structure, macrocycle, synchrotron radiation, silver(II) complex, nitrate ion, *trans*-III conformation, hydrogen bonding

## Abstract

The asymmetric unit of the title compound, [Ag(C_24_H_46_N_6_O_2_)](NO_3_)_2_ [C_24_H_46_N_6_O_2_ is (5,16-dimethyl-2,6,13,17-tetra­aza­tri­cyclo­[16.4.0.0^7,12^]docosane-2,13-di­yl)diacetamide, *L*], consists of one independent half of the [Ag(C_24_H_46_N_6_O_2_)]^2+^ cation and one nitrate anion. The Ag atom, lying on an inversion centre, has a square-planar geometry and the complex adopts a stable *trans*-III conformation. Inter­estingly, the two O atoms of the pendant acetamide groups are not coordinated to the Ag^II^ ion. The longer distance of 2.227 (2) Å for Ag—N(tertiary) compared to 2.134 (2) Å for Ag—N(secondary) may be due to the effects of the attached acetamide group on the tertiary N atom. Two nitrate anions are very weakly bound to the Ag^II^ ion in the axial sites and are further connected to the ligand of the cation by N—H⋯O hydrogen bonds. The crystal packing is stabilized by hydrogen-bonding inter­actions among the N—H donor groups of the macrocycle and its actetamide substituents, and the O atoms of the nitrate anions and of an acetamide group as the acceptor atoms.

## Abstract 2

The title compound, [Ag(C_24_H_46_N_6_O_2_)](NO_3_)_2_, has a square-planar geometry with the nitrate anions on general sites. The macrocycle adopts the *trans*-III conformation. The crystal packing is stabilized by hydrogen-bonding inter­actions among the N–H groups of the macrocycle and its actetamide substituents, with the O atoms of the nitrate anions and of an acetamide group as the acceptor atoms.

## Chemical context   

Macrocycles with *N*-substituted groups on the polyaza macrocyclic ring and their transition metal complexes have attracted considerable attention because of their structural and chemical properties, which are different from those of the corresponding unsubstituted macrocyclic systems. Recently, it has been shown that the cyclam (1,4,8,11-tetra­aza­cyclo­tetra­deca­ne) derivatives and their metal complexes exhibit anti-HIV activity (Ronconi & Sadler, 2007 ▸; De Clercq, 2010 ▸; Ross *et al.*, 2012 ▸). These cyclam-based macrocyclic ligands have a moderately flexible structure, and can adopt both planar (*trans*) and folded (*cis*) configurations. There are five conformational *trans* isomers for the cyclam moiety, which differ in the chirality of the *sec*-NH centers (Choi, 2009 ▸). The *trans*-I, *trans*-II and *trans*-V configurations can fold to form *cis*-I, *cis*-II and *cis*-V isomers, respectively (Subhan *et al.*, 2011 ▸). The conformation of the macrocyclic ligand and the orientations of the N–H bonds are very important factors for co-receptor recognition. Therefore, knowledge of the conformation and crystal packing of transition metal complexes containing the cyclam ligand has become important in the development of new highly effective anti-HIV drugs that specially target alternative events in the HIV replicative cycle (De Clercq, 2010 ▸). Partially *N*-substituted tetra­aza­macrocycles and their complexes have been much less widely studied. This may be due to the difficulty encountered in the attachment of only one or two pendant arms to the tetra­aza macrocycle by several steps and in low yields. The presence of two methyl substit­uents on the macrocyclic ring carbon atoms next to the secondary amine groups facilitates syntheses, as *N*-substitution takes place only on the less sterically hindered nitro­gen atoms.
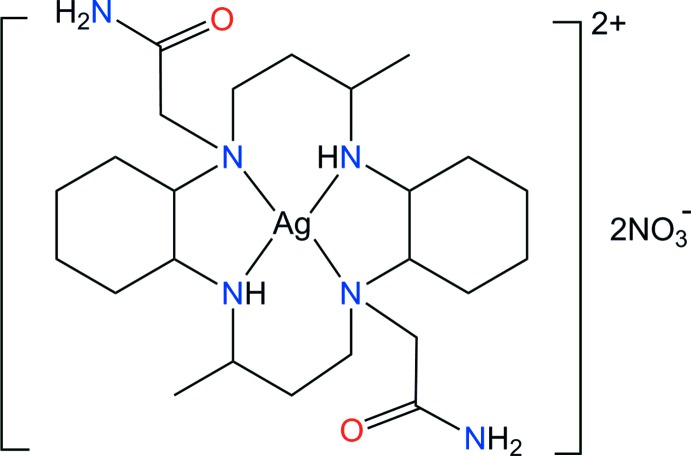


The syntheses and crystal structures of transition metal complexes with the constrained cyclam ligand containing two acetamide groups on the nitro­gen atoms have received much attention because of the effects of the functional groups on their chemical properties and coordination geometry (Choi *et al.*, 2001*a* ▸,*b* ▸,*c* ▸; Choi & Lee, 2007 ▸). The nitrate ion can also coordinate to the transition metal ions in a monodentate, chelating bidentate or bridging bidentate fashion. The oxidation state of the metal, the nature of other ligands and steric factors influence the mode of coordination.

In this communication, we report the synthesis and structural characterization a new silver(II) complex, [Ag(C_24_H_46_N_6_O_2_)](NO_3_)_2_, (I) to confirm the conformation and bonding modes of the macrocyclic ligand and the nitrate anions.

## Structural commentary   

**Figure 1:**
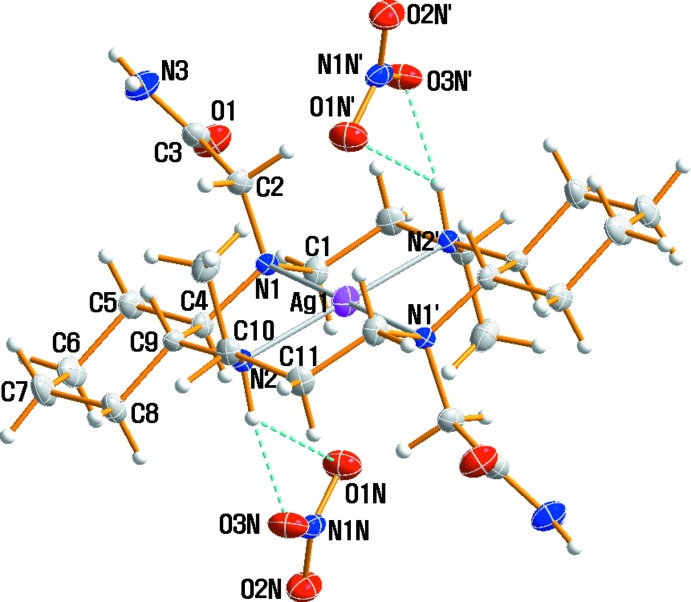
A perspective view (50% probability) of complex (I). The primed atoms are related by the symmetry operation (−*x* + 1, −*y* + 1, −*z* + 1). Hydrogen bonds are drawn as dashed lines.

The structural analysis showed the space group to be *P* 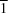 with *Z* = 1. The asymmetric unit contains one independent half of the [Ag(C_24_H_46_N_6_O_2_)]^2+^ cation and one nitrate anion. The silver(II) cation is situated on a center of inversion in the small triclinic cell, which contains a single silver(II) complex. An ellipsoid plot of the title compound is shown in Fig. 1 ▸ along with the atomic numbering scheme. The two methyl groups on the six-membered chelate rings and the two –(CH_2_)_4_– parts of the cyclo­hexane backbones are *anti* with respect to the macrocyclic plane. Two pendant acetamide groups in the Ag^II^ complex mol­ecule are also *trans* to each other, and thus the macrocyclic skeleton adopts the most stable *trans*-III (RRSS) conformation. The five-membered chelate rings adopt a *gauche*, and the six-membered rings are in chair conformations. The Ag^II^ cation is surrounded by a square-planar array of four nitro­gen atoms from the secondary and tertiary amines in the macrocycle. Inter­estingly, the oxygen atoms of the acetamide substituents are not coordinated to the metal center. It is noteworthy that the Zn^II^, Ni^II^ and Cu^II^ complexes of the same ligand have a tetra­gonally distorted octa­hedral environment with the four N atoms of the macrocyclic ligand in equatorial positions and the O atoms of the pendant acetamide groups in axial positions (Choi *et al.*, 2001*a* ▸,*b* ▸,*c* ▸; Choi & Lee, 2007 ▸). The Ag—N bond lengths of 2.134 (2) and 2.227 (2) Å from the donor atoms of the macrocycle can be compared to those determined in [Ag(cyclam)](ClO_4_)_2_ [2.158 (2)–2.192 (2) Å; Ito *et al.*, 1981 ▸], [Ag(tmc)](ClO_4_)_2_ [2.194 (2)–2.196 (2) Å; tmc = 1,4,8,11-tetra­methyl-1,4,8,11-tetra­aza­cyclo­tetra­decane; Po *et al.*, 1991 ▸], [Ag(tet *a*)](NO_3_)_2_ [2.159 (3)–2.162 (3) Å; tet *a* = *C*-*meso*-5,5,7,12,12,14-hexa­methyl-1,4,8,11-tetra­aza­cyclo­tetra­decane; Mertes, 1978 ▸] and [Ag(3,14-dimethyl-2,6,13,17-tetra­aza­tri­cyclo­[16.4.0.0^7,12^]doco­sa­ne)](NO_3_)_2_·4H_2_O [2.140 (2)–2.150 (3) Å; Moon *et al.*, 2010 ▸]. The longer Ag—N(tertiary) bond distance, compared to the length of the Ag—N(secondary) bond may be due to the steric and inductive effects of the pendant acetamide group on the tertiary N atom. The Ag—O distance of 3.109 (2) Å is longer than the corresponding distances in [Ag(cyclam)](ClO_4_)_2_ [2.788 (2) Å; Ito *et al.*, 1981 ▸], [Ag(tmc)](ClO_4_)_2_ [2.889 (4) Å; Po *et al.*, 1991 ▸], [Ag(tet *a*)](NO_3_)_2_ [2.807 (4) Å; Mertes, 1978 ▸] and [Ag(3,14-dimethyl-2,6,13,17-tetra­aza­tri­cyclo­[16.4.0.0^7,12^]docosa­ne)](NO_3_)_2_·4H_2_O [2.923 (2) Å; Moon *et al.*, 2010 ▸]. The longest N1—C4 bond distance is also probably due to the effect of the acetamide group and the cyclo­hexane ring. The nitrate anion has a slightly distorted trigonal-planar geometry because of the hydrogen bonding inter­actions and the very weak inter­action with the silver(II) ion. Two nitrate ions are located above and below the coordination planes, and each are linked to the cation *via* N—H⋯O hydrogen bonds.

## Supra­molecular features   

**Figure 2:**
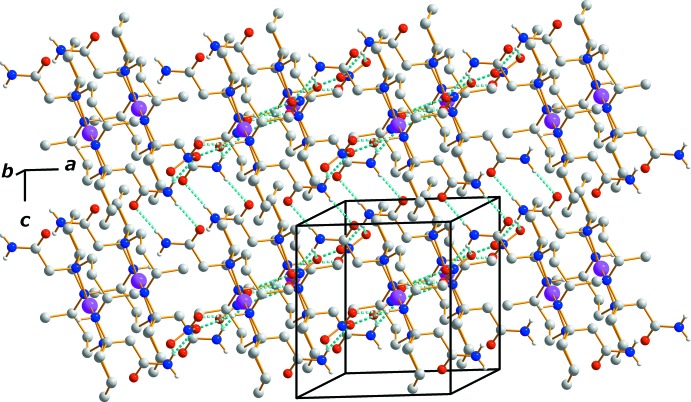
The crystal packing in complex (I), viewed along the *b*-axis direction. Dashed lines represent N—H⋯O hydrogen-bonding inter­actions.

Extensive hydrogen-bonding inter­actions occur in the crystal structure (Table 1 ▸). The nitrate ions are connected to the ligand of the cation *via* N—H⋯O hydrogen bonds. The nitrate anions have slightly distorted trigonal–planar geometries because of these inter­actions and the very weak inter­action with the silver(II) cation. The supra­molecular architecture involves hydrogen bonds between the N—H groups of both the macrocycle and its pendant acetamide substituents as donors, and the O atoms of the nitrate anions and the acetamides as acceptors. An array of these contacts generate a two-dimensional sheet of mol­ecules stacked along the *b-*axis direction (Fig. 2 ▸). This hydrogen-bonded network helps to stabilize the crystal structure.

## Database survey   

A search of the Cambridge Structural Database (Version 5.38, May 2017 with three updates; Groom *et al.*, 2016 ▸) gave four hits for the macrocycle (C_24_H_46_N_6_O_2_) unit. The crystal structures of [Cu(C_24_H_46_N_6_O_2_)]Cl_2_·8H_2_O (Choi *et al.*, 2001*a* ▸), [Zn(C_24_H_46_N_6_O_2_)]Cl_2_·3H_2_O (Choi *et al.*, 2001*b* ▸), [Ni(C_24_H_46_N_6_O_2_)](ClO_4_)_2_ (Choi *et al.*, 2001*c* ▸) and [Cu(C_24_H_46_N_6_O_2_)](ClO_4_)_2_ (Choi *et al.*, 2001*c* ▸) have been reported previously. In all of these structures, two O atoms of the acetamide substit­uents occupy the axial positions, giving rise to a tetra­gonally distorted octa­hedral geometry. This is quite unlike the square-planar geometry of the title compound as the two O atoms of the acetamide substituents are not bound to the silver(II) cation in this case. Until now, no structure of the complex ion [Ag(C_24_H_46_N_6_O_2_)]^2+^ with any anion has been reported.

## Synthesis and crystallization   

As a starting material, 3,14-dimethyl-2,6,13,17-tetra­aza­tri­cyclo­[16.4.0.0^7,12^]docosane was prepared according to a published procedure (Kang *et al.*, 1991 ▸). All other chemicals were purchased from commercial sources and used without further purification. The macrocyclic ligand 2,13-bis­(acet­amido)-5,16-dimethyl-2,6,13,17-tetra­aza­tri­cyclo­[16.4.0.0^7,12^]docosane (*L*) was prepared by a previously reported method (Maumela *et al.*, 1995 ▸). AgNO**_3_** (0.34 g, 2 mmol) dissolved in water (10 mL) was mixed with a suspension of the ligand *L* (0.45 g, 1 mmol) in methanol (20 mL). The resulting mixture was heated at 313 K for 30 min and then filtered to remove metallic silver. The orange filtrate was left in an open beaker, protected from the light, at ambient temperature. After several days block-like dark-orange crystals of (I) suitable only for synchrotron X-ray analysis were formed.

In the synthesis of the title complex, two pertinent features are found. One is that the complex contains the silver in the unusually high oxidation state, Ag^II^. This is stabilized by the macrocycle *L*. The complex is the product of the disproportionation of the Ag^I^ complex according to the following equation:

2Ag^I^ + *L* → Ag^II^ *L* + Ag(*s*) ↓

It is generally understood that macrocyclic ligands possess a suitable cavity size and hard nitro­gen donor atoms that can form stable Ag^II^ complexes in aqueous solution (Ali *et al.*, 2004 ▸).

## Refinement   

Crystal data, data collection and structure refinement details are summarized in Table 2 ▸. All H atoms were placed in geometrically idealized positions and constrained to ride on their parent atoms, with C—H distances of 0.98–1.00 Å and an N—H distance of 0.88–1.0 Å. All displacement parameters of H atoms *U*_iso_(H) were set to 1.2 or 1.5*U*_eq_ of their respective parent atoms.

## Supplementary Material

Crystal structure: contains datablock(s) I. DOI: 10.1107/S2056989018003560/sj5548sup1.cif

Structure factors: contains datablock(s) I. DOI: 10.1107/S2056989018003560/sj5548Isup2.hkl

CCDC reference: 1826672

Additional supporting information:  crystallographic information; 3D view; checkCIF report

## Figures and Tables

**Table 1:** Hydrogen-bond geometry (Å, °)

*D*—H⋯*A*	*D*—H	H⋯*A*	*D*⋯*A*	*D*—H⋯*A*
N2—H2⋯O1*N*	1.00	2.59	3.214 (4)	121
N2—H2⋯O3*N*	1.00	1.93	2.925 (4)	172
N3—H3*A*⋯O1^i^	0.88	2.03	2.913 (4)	177
N3—H3*B*⋯O1*N* ^ii^	0.88	2.06	2.930 (4)	168
N3—H3*B*⋯O2*N* ^ii^	0.88	2.59	3.281 (4)	136

Symmetry codes: (i) 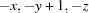; (ii) 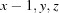.

**Table 2:** Experimental details

Crystal data	
Chemical formula	[Ag(C_24_H_46_N_6_O_2_)](NO_3_)_2_
*M*_r_	682.56
Crystal system, space group	Triclinic, *P* 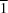
Temperature (K)	173
*a*, *b*, *c* (Å)	8.3460 (17), 9.2874 (19), 10.171 (2)
α, β, γ (°)	104.32 (3), 90.28 (3), 109.60 (3)
*V* (Å^3^)	716.3 (3)
*Z*	1
Radiation type	Synchrotron, λ = 0.610 Å
μ (mm^−1^)	0.51
Crystal size (mm)	0.02 × 0.02 × 0.01

Data collection	
Diffractometer	ADSC Q210 CCD area detector
Absorption correction	Empirical (using intensity measurements) (*HKL3000sm *SCALEPACK**; Otwinowski & Minor, 1997 ▸)
*T*_min_, *T*_max_	0.937, 1.000
No. of measured, independent and observed [*I* > 2σ(*I*)] reflections	7431, 3750, 3418
*R*_int_	0.034
(sin θ/λ)_max_ (Å^−1^)	0.693

Refinement	
*R*[*F* ^2^ > 2σ(*F* ^2^)], *wR*(*F* ^2^), *S*	0.043, 0.115, 1.05
No. of reflections	3750
No. of parameters	189
H-atom treatment	H-atom parameters constrained
Δρ_max_, Δρ_min_ (e Å^−3^)	0.71, −2.17

Computer programs: *PAL BL2D-SMDC Program* (Shin *et al., * 2016 ▸), *HKL3000sm* (Otwinowski & Minor, 1997 ▸), *SHELXT2014* (Sheldrick, 2015*a* ▸), *SHELXL2018* (Sheldrick, 2015*b* ▸), *DIAMOND 4* (Putz & Brandenburg, 2014 ▸) and *publCIF* (Westrip, 2010 ▸).

## supplementary crystallographic information

### Crystal data

**Table:**

[Ag(C_24_H_46_N_6_O_2_)](NO_3_)_2_	*Z* = 1
*M**_r_* = 682.56	*F*(000) = 357
Triclinic, *P*1	*D*_x_ = 1.582 Mg m^−^^3^
*a* = 8.3460 (17) Å	Synchrotron radiation, λ = 0.610 Å
*b* = 9.2874 (19) Å	Cell parameters from 46429 reflections
*c* = 10.171 (2) Å	θ = 0.4–33.7°
α = 104.32 (3)°	µ = 0.51 mm^−^^1^
β = 90.28 (3)°	*T* = 173 K
γ = 109.60 (3)°	Block, dark orange
*V* = 716.3 (3) Å^3^	0.02 × 0.02 × 0.01 mm

### Data collection

**Table:**

ADSC Q210 CCD area detector diffractometer	3418 reflections with *I* > 2σ(*I*)
Radiation source: PLSII 2D bending magnet	*R*_int_ = 0.034
ω scan	θ_max_ = 25.0°, θ_min_ = 1.8°
Absorption correction: empirical (using intensity measurements) (*HKL3000sm SCALEPACK*; Otwinowski & Minor, 1997)	*h* = −11→11
*T*_min_ = 0.937, *T*_max_ = 1.000	*k* = −12→12
7431 measured reflections	*l* = −14→14
3750 independent reflections	

### Refinement

**Table:**

Refinement on *F*^2^	Hydrogen site location: inferred from neighbouring sites
Least-squares matrix: full	H-atom parameters constrained
*R*[*F*^2^ > 2σ(*F*^2^)] = 0.043	*w* = 1/[σ^2^(*F*_o_^2^) + (0.0747*P*)^2^] where *P* = (*F*_o_^2^ + 2*F*_c_^2^)/3
*wR*(*F*^2^) = 0.115	(Δ/σ)_max_ < 0.001
*S* = 1.05	Δρ_max_ = 0.71 e Å^−^^3^
3750 reflections	Δρ_min_ = −2.17 e Å^−^^3^
189 parameters	Extinction correction: SHELXL2018 (Sheldrick, 2015b), Fc^*^=kFc[1+0.001xFc^2^λ^3^/sin(2θ)]^-1/4^
0 restraints	Extinction coefficient: 0.065 (6)

### Special details

**Table:**

Geometry. All esds (except the esd in the dihedral angle between two l.s. planes) are estimated using the full covariance matrix. The cell esds are taken into account individually in the estimation of esds in distances, angles and torsion angles; correlations between esds in cell parameters are only used when they are defined by crystal symmetry. An approximate (isotropic) treatment of cell esds is used for estimating esds involving l.s. planes.

### Fractional atomic coordinates and isotropic or equivalent isotropic displacement parameters (Å^2^)

**Table:**

	*x*	*y*	*z*	*U*_iso_*/*U*_eq_	
Ag1	0.500000	0.500000	0.500000	0.01559 (13)	
O1	0.2273 (3)	0.5401 (3)	0.0700 (3)	0.0332 (6)	
N1	0.3945 (3)	0.4502 (3)	0.2855 (2)	0.0138 (4)	
N2	0.3842 (3)	0.2496 (3)	0.4657 (2)	0.0141 (4)	
H2	0.480698	0.207953	0.460293	0.017*	
N3	−0.0231 (4)	0.4445 (4)	0.1558 (3)	0.0287 (6)	
H3A	−0.082643	0.448023	0.085766	0.034*	
H3B	−0.075649	0.410273	0.222782	0.034*	
C1	0.5320 (4)	0.5522 (4)	0.2206 (3)	0.0190 (6)	
H1A	0.491733	0.531956	0.123804	0.023*	
H1AB	0.633336	0.519689	0.222357	0.023*	
C2	0.2334 (4)	0.4871 (4)	0.2920 (3)	0.0200 (6)	
H2A	0.258145	0.591883	0.357970	0.024*	
H2AB	0.149558	0.408176	0.330418	0.024*	
C3	0.1459 (4)	0.4909 (4)	0.1604 (3)	0.0223 (6)	
C4	0.3680 (4)	0.2774 (3)	0.2288 (3)	0.0167 (5)	
H4	0.483624	0.268837	0.217142	0.020*	
C5	0.2651 (4)	0.2038 (4)	0.0888 (3)	0.0218 (6)	
H5A	0.148066	0.206799	0.096639	0.026*	
H5B	0.319617	0.266303	0.024804	0.026*	
C6	0.2561 (5)	0.0326 (4)	0.0329 (3)	0.0296 (7)	
H6A	0.186557	−0.013650	−0.056301	0.035*	
H6B	0.372603	0.030416	0.018612	0.035*	
C7	0.1776 (5)	−0.0665 (4)	0.1307 (3)	0.0318 (8)	
H7A	0.179869	−0.175056	0.095173	0.038*	
H7B	0.056864	−0.074055	0.137454	0.038*	
C8	0.2763 (5)	0.0082 (4)	0.2717 (3)	0.0249 (6)	
H8A	0.393756	0.005616	0.265973	0.030*	
H8B	0.220186	−0.054797	0.334862	0.030*	
C9	0.2852 (4)	0.1794 (3)	0.3284 (3)	0.0148 (5)	
H9	0.166364	0.180502	0.337656	0.018*	
C10	0.2906 (4)	0.2024 (3)	0.5819 (3)	0.0182 (5)	
H10	0.252172	0.084275	0.560973	0.022*	
C11	0.4128 (4)	0.2696 (4)	0.7127 (3)	0.0207 (6)	
H11A	0.357944	0.213455	0.780635	0.025*	
H11B	0.517100	0.243381	0.692138	0.025*	
C12	0.1312 (4)	0.2481 (4)	0.5968 (3)	0.0269 (7)	
H12A	0.159371	0.357672	0.591454	0.040*	
H12B	0.088753	0.238874	0.685098	0.040*	
H12C	0.042823	0.177157	0.523376	0.040*	
O1N	0.7629 (4)	0.3507 (3)	0.3690 (3)	0.0343 (6)	
O2N	0.8673 (3)	0.1611 (3)	0.3122 (3)	0.0338 (6)	
O3N	0.6869 (3)	0.1576 (3)	0.4643 (3)	0.0328 (6)	
N1N	0.7737 (3)	0.2225 (3)	0.3815 (3)	0.0237 (5)	

### Atomic displacement parameters (Å^2^)

**Table:**

	*U*^11^	*U*^22^	*U*^33^	*U*^12^	*U*^13^	*U*^23^
Ag1	0.01841 (18)	0.01836 (19)	0.00929 (16)	0.00500 (12)	−0.00143 (10)	0.00443 (10)
O1	0.0323 (13)	0.0502 (16)	0.0254 (12)	0.0174 (12)	0.0029 (10)	0.0203 (11)
N1	0.0189 (11)	0.0161 (11)	0.0080 (9)	0.0073 (9)	−0.0004 (8)	0.0043 (8)
N2	0.0177 (11)	0.0149 (11)	0.0100 (10)	0.0054 (9)	0.0004 (8)	0.0042 (8)
N3	0.0271 (14)	0.0434 (18)	0.0213 (13)	0.0161 (13)	0.0001 (11)	0.0132 (12)
C1	0.0216 (14)	0.0232 (14)	0.0102 (11)	0.0044 (11)	0.0030 (10)	0.0058 (10)
C2	0.0248 (15)	0.0243 (15)	0.0128 (12)	0.0118 (12)	−0.0022 (10)	0.0038 (10)
C3	0.0306 (16)	0.0216 (15)	0.0172 (13)	0.0127 (13)	−0.0030 (11)	0.0045 (11)
C4	0.0200 (13)	0.0201 (14)	0.0084 (11)	0.0068 (11)	−0.0012 (9)	0.0013 (9)
C5	0.0291 (16)	0.0251 (15)	0.0103 (12)	0.0110 (13)	−0.0036 (10)	0.0007 (10)
C6	0.0408 (19)	0.0286 (17)	0.0162 (14)	0.0164 (15)	−0.0059 (13)	−0.0056 (12)
C7	0.044 (2)	0.0203 (16)	0.0237 (16)	0.0103 (15)	−0.0127 (14)	−0.0049 (12)
C8	0.0338 (17)	0.0178 (14)	0.0194 (14)	0.0074 (13)	−0.0060 (12)	0.0006 (11)
C9	0.0177 (13)	0.0165 (13)	0.0112 (11)	0.0079 (10)	−0.0011 (9)	0.0028 (9)
C10	0.0211 (14)	0.0175 (13)	0.0153 (12)	0.0036 (11)	0.0023 (10)	0.0073 (10)
C11	0.0275 (15)	0.0233 (15)	0.0136 (12)	0.0078 (12)	0.0017 (11)	0.0106 (11)
C12	0.0206 (15)	0.0370 (19)	0.0193 (14)	0.0072 (13)	0.0040 (11)	0.0046 (13)
O1N	0.0386 (15)	0.0353 (14)	0.0385 (14)	0.0187 (12)	0.0114 (11)	0.0183 (11)
O2N	0.0326 (14)	0.0448 (16)	0.0263 (12)	0.0211 (12)	0.0051 (10)	0.0027 (11)
O3N	0.0376 (14)	0.0359 (14)	0.0388 (14)	0.0232 (12)	0.0152 (11)	0.0195 (11)
N1N	0.0222 (13)	0.0297 (15)	0.0203 (12)	0.0137 (11)	−0.0037 (10)	0.0021 (10)

### Geometric parameters (Å, º)

**Table:**

Ag1—N2^i^	2.134 (2)	C5—H5A	0.9900
Ag1—N2	2.134 (2)	C5—H5B	0.9900
Ag1—N1	2.227 (2)	C6—C7	1.523 (5)
Ag1—N1^i^	2.227 (2)	C6—H6A	0.9900
O1—C3	1.234 (4)	C6—H6B	0.9900
N1—C2	1.493 (4)	C7—C8	1.527 (4)
N1—C1	1.496 (4)	C7—H7A	0.9900
N1—C4	1.504 (4)	C7—H7B	0.9900
N2—C9	1.494 (3)	C8—C9	1.527 (4)
N2—C10	1.495 (3)	C8—H8A	0.9900
N2—H2	1.0000	C8—H8B	0.9900
N3—C3	1.326 (4)	C9—H9	1.0000
N3—H3A	0.8800	C10—C12	1.524 (4)
N3—H3B	0.8800	C10—C11	1.532 (4)
C1—C11^i^	1.530 (4)	C10—H10	1.0000
C1—H1A	0.9900	C11—H11A	0.9900
C1—H1AB	0.9900	C11—H11B	0.9900
C2—C3	1.535 (4)	C12—H12A	0.9800
C2—H2A	0.9900	C12—H12B	0.9800
C2—H2AB	0.9900	C12—H12C	0.9800
C4—C5	1.531 (4)	O1N—N1N	1.261 (4)
C4—C9	1.540 (4)	O2N—N1N	1.240 (4)
C4—H4	1.0000	O3N—N1N	1.249 (4)
C5—C6	1.526 (5)		
			
N2^i^—Ag1—N2	180.0	H5A—C5—H5B	108.1
N2^i^—Ag1—N1	96.57 (9)	C7—C6—C5	111.1 (3)
N2—Ag1—N1	83.43 (9)	C7—C6—H6A	109.4
N2^i^—Ag1—N1^i^	83.43 (9)	C5—C6—H6A	109.4
N2—Ag1—N1^i^	96.57 (9)	C7—C6—H6B	109.4
N1—Ag1—N1^i^	180.0 (2)	C5—C6—H6B	109.4
C2—N1—C1	114.7 (2)	H6A—C6—H6B	108.0
C2—N1—C4	114.1 (2)	C6—C7—C8	110.4 (3)
C1—N1—C4	111.6 (2)	C6—C7—H7A	109.6
C2—N1—Ag1	106.66 (16)	C8—C7—H7A	109.6
C1—N1—Ag1	105.59 (17)	C6—C7—H7B	109.6
C4—N1—Ag1	102.92 (15)	C8—C7—H7B	109.6
C9—N2—C10	115.8 (2)	H7A—C7—H7B	108.1
C9—N2—Ag1	109.76 (16)	C7—C8—C9	111.9 (3)
C10—N2—Ag1	113.18 (17)	C7—C8—H8A	109.2
C9—N2—H2	105.7	C9—C8—H8A	109.2
C10—N2—H2	105.7	C7—C8—H8B	109.2
Ag1—N2—H2	105.7	C9—C8—H8B	109.2
C3—N3—H3A	120.0	H8A—C8—H8B	107.9
C3—N3—H3B	120.0	N2—C9—C8	110.5 (2)
H3A—N3—H3B	120.0	N2—C9—C4	110.5 (2)
N1—C1—C11^i^	115.2 (2)	C8—C9—C4	109.6 (2)
N1—C1—H1A	108.5	N2—C9—H9	108.7
C11^i^—C1—H1A	108.5	C8—C9—H9	108.7
N1—C1—H1AB	108.5	C4—C9—H9	108.7
C11^i^—C1—H1AB	108.5	N2—C10—C12	111.9 (2)
H1A—C1—H1AB	107.5	N2—C10—C11	109.9 (2)
N1—C2—C3	119.0 (2)	C12—C10—C11	113.0 (2)
N1—C2—H2A	107.6	N2—C10—H10	107.3
C3—C2—H2A	107.6	C12—C10—H10	107.3
N1—C2—H2AB	107.6	C11—C10—H10	107.3
C3—C2—H2AB	107.6	C1^i^—C11—C10	117.6 (2)
H2A—C2—H2AB	107.0	C1^i^—C11—H11A	107.9
O1—C3—N3	123.2 (3)	C10—C11—H11A	107.9
O1—C3—C2	122.4 (3)	C1^i^—C11—H11B	107.9
N3—C3—C2	114.3 (3)	C10—C11—H11B	107.9
N1—C4—C5	113.8 (2)	H11A—C11—H11B	107.2
N1—C4—C9	111.8 (2)	C10—C12—H12A	109.5
C5—C4—C9	109.8 (2)	C10—C12—H12B	109.5
N1—C4—H4	107.0	H12A—C12—H12B	109.5
C5—C4—H4	107.0	C10—C12—H12C	109.5
C9—C4—H4	107.0	H12A—C12—H12C	109.5
C6—C5—C4	110.7 (3)	H12B—C12—H12C	109.5
C6—C5—H5A	109.5	O2N—N1N—O3N	120.6 (3)
C4—C5—H5A	109.5	O2N—N1N—O1N	120.7 (3)
C6—C5—H5B	109.5	O3N—N1N—O1N	118.6 (3)
C4—C5—H5B	109.5		
			
C2—N1—C1—C11^i^	57.5 (3)	C6—C7—C8—C9	56.2 (4)
C4—N1—C1—C11^i^	−170.8 (2)	C10—N2—C9—C8	−78.2 (3)
Ag1—N1—C1—C11^i^	−59.7 (3)	Ag1—N2—C9—C8	152.1 (2)
C1—N1—C2—C3	53.5 (3)	C10—N2—C9—C4	160.3 (2)
C4—N1—C2—C3	−77.0 (3)	Ag1—N2—C9—C4	30.6 (3)
Ag1—N1—C2—C3	170.0 (2)	C7—C8—C9—N2	−179.4 (3)
N1—C2—C3—O1	−35.8 (4)	C7—C8—C9—C4	−57.4 (4)
N1—C2—C3—N3	147.4 (3)	N1—C4—C9—N2	−52.8 (3)
C2—N1—C4—C5	54.2 (3)	C5—C4—C9—N2	179.9 (2)
C1—N1—C4—C5	−77.8 (3)	N1—C4—C9—C8	−174.8 (2)
Ag1—N1—C4—C5	169.4 (2)	C5—C4—C9—C8	57.9 (3)
C2—N1—C4—C9	−70.9 (3)	C9—N2—C10—C12	−59.3 (3)
C1—N1—C4—C9	157.1 (2)	Ag1—N2—C10—C12	68.7 (3)
Ag1—N1—C4—C9	44.3 (2)	C9—N2—C10—C11	174.3 (2)
N1—C4—C5—C6	175.4 (3)	Ag1—N2—C10—C11	−57.7 (3)
C9—C4—C5—C6	−58.5 (3)	N2—C10—C11—C1^i^	73.2 (3)
C4—C5—C6—C7	57.5 (4)	C12—C10—C11—C1^i^	−52.5 (3)
C5—C6—C7—C8	−55.6 (4)		

Symmetry code: (i) −*x*+1, −*y*+1, −*z*+1.

### Hydrogen-bond geometry (Å, º)

**Table:**

*D*—H···*A*	*D*—H	H···*A*	*D*···*A*	*D*—H···*A*
N2—H2···O1*N*	1.00	2.59	3.214 (4)	121
N2—H2···O3*N*	1.00	1.93	2.925 (4)	172
N3—H3*A*···O1^ii^	0.88	2.03	2.913 (4)	177
N3—H3*B*···O1*N*^iii^	0.88	2.06	2.930 (4)	168
N3—H3*B*···O2*N*^iii^	0.88	2.59	3.281 (4)	136

Symmetry codes: (ii) −*x*, −*y*+1, −*z*; (iii) *x*−1, *y*, *z*.
